# Vardenafil-Loaded Bilosomal Mucoadhesive Sponge for Buccal Delivery: Optimization, Characterization, and In Vivo Evaluation

**DOI:** 10.3390/polym14194184

**Published:** 2022-10-05

**Authors:** Mohammed F. Aldawsari, El-Sayed Khafagy, Hadil Faris Alotaibi, Amr Selim Abu Lila

**Affiliations:** 1 Department of Pharmaceutics, College of Pharmacy, Prince Sattam Bin Abdulaziz University, Al-kharj 11942, Saudi Arabia; 2 Department of Pharmaceutics and Industrial Pharmacy, Faculty of Pharmacy, Suez Canal University, Ismailia 41522, Egypt; 3 Department of Pharmaceutical Sciences, College of Pharmacy, Princess Nourah Bint Abdulrahman University, Riyadh 11671, Saudi Arabia; 4 Department of Pharmaceutics, College of Pharmacy, University of Hail, Hail 81442, Saudi Arabia; 5 Department of Pharmaceutics and Industrial Pharmacy, Faculty of Pharmacy, Zagazig University, Zagazig 44519, Egypt

**Keywords:** bilosomes, buccal delivery, cGMP, mucoadhesive sponge, vardenafil

## Abstract

Vardenafil (VDF) is a relatively new phosphodiesterase-5 inhibitor that has limited oral bioavailability (≈15%). The objective of this study was to develop bilosome-based mucoadhesive buccal sponge for augmenting the oral bioavailability of VDF. VDF-loaded bilosomes were fabricated and optimized using a Box-Behnken design. The optimized VDF-loaded bilosomal formulation was assessed for surface morphology, particle size, thermal characteristics, and in vitro release. Afterwards, the optimized bilosomal formulation was incorporated into a cellulose-based matrix to obtain buccal sponge, which was evaluated for ex vivo permeation studies, in vivo oral bioavailability, and in vivo serum concentration of cyclic guanosine monophosphate (cGMP). The mean particle size and entrapment efficiency (%) of optimized bilosome formulation were 282.6 ± 9.5 nm and 82.95 ± 3.5%, respectively. In vitro release studies at pH 6.8 emphasized the potential of optimized bilosomal formulation to sustain VDF release for 12 h. Ex vivo permeation study using sheep buccal mucosa indicated significant enhancement in penetration of VDF from bilosomal buccal sponge compared to plain VDF gel. Pharmacokinetic study in Albino rats showed ~5 fold increase in relative bioavailability with bilosomal buccal sponge, compared to VDF suspension. In addition, VDF-loaded bilosomal buccal sponge triggered higher serum levels of cGMP, a biomarker of VDF in vivo efficacy, compared to oral VDF suspension. To sum up, bilosomes might represent a potential nanocarrier for buccal delivery of VDF, enhancing its oral bioavailability and therapeutic efficacy.

## 1. Introduction

Vardenafil (VDF) is a highly selective oral phosphodiesterase type 5 (PDE-5) inhibitor [1]. It competitively inhibits cyclic guanosine monophosphate (cGMP) degradation by phosphodiesterase-5 enzyme, promoting cGMP accumulation, resulting in vascular smooth muscle relaxation and vasodilation [2]. VDF has been initially prescribed for the treatment of arterial pulmonary hypertension and, more recently, for the management of erectile dysfunction [3,4]. Nevertheless, VDF shows limited systemic bioavailability due to substantial first-pass metabolism and limited water solubility; VDF is classified as a class II (high permeability/low solubility) drug in the Biopharmaceutics Classification System (BCS). Accordingly, it is crucial to develop a dosage form that could improve VDF solubility while bypassing first-pass effect. 

Recently, there has been an increased interest in employing nanocarrier systems for oral delivery since they provide various advantages over traditional dosage forms [5], such as enhancing the solubility rates of poorly soluble drugs, efficiently bypassing first-pass metabolism, which collectively result in higher drug bioavailability [6,7]. Among various nanocarrier systems, lipid-based nanocarrier systems have surged as potential delivery vehicles for improving the solubility, bioavailability, and/or stability of lipophilic drugs [7,8,9,10]. In particular, niosomes and liposomes have shown the ability to entrap both hydrophilic and lipophilic drugs [11], as well as to shield the entrapped drug from being degraded by gastrointestinal tract (GIT) enzymes [12]. Nevertheless, the major problems with traditional nano-vesicular carriers were drug leakage and storage instability.

Instead, bilosomes, bilayered vesicles stabilized by bile salts, have recently been proposed as an alternative to the commonly used conventional vesicular systems (niosomes and liposomes). Bilosomes are lipid nanovesicles enriched with bile salts that have a high potential to cross biological membranes [13]. The incorporation of bile salts into the structure of bilosomes has been reported to limit the degradation of nanovesicles in the GIT, enhance penetration and make oral delivery more effective [14]. In addition, the existence of negatively charged bile salts such as sodium deoxycholate was found to enhance the colloidal stability of bilosomes [7,13]. Most importantly, by virtue of its small nano-size range and its fluidizing action, bilosomes could grant efficient permeability through biological barriers, and thereby, augment drug absorption [15].

The buccal cavity has been extensively studied as an alternative to the oral route for drug delivery [16,17]. The buccal route offers various benefits over oral administration, including bypassing hepatic metabolism and pre-systemic clearance from GIT [18]. A wide range of dosage forms (tablets, films, gels, sponges, etc.) for buccal administration have recently been developed and marketed [19,20]. Among them, mucoadhesive sponges have demonstrated the merits of permitting longer drug residence within the buccal cavity for more effective drug absorption [7,21,22].

The objective of the current study, therefore, was to develop a novel VDF-loaded bilosomal formulation using a Box-Behnken design followed by numerical optimization. The obtained optimized bilosomal formulation was characterized and then incorporated into cellulose-based matrix to form a mucoadhesive buccal formulation. The in vivo efficiency of such novel VDF-loaded bilosomal mucoadhesive buccal formulation was evaluated via pharmacokinetic studies and pharmacodynamic assessment of cGMP serum level. The novelty of this study comprises the use of bilosomes, for the first time, as an evolving vesicular carrier system for enhancing the oral bioavailability of VDF. Another unique characteristic of this research is the stabilization of VDF-loaded bilosomes through integration into buccal mucoadhesive sponges for the purpose of permitting better drug absorption.

## 2. Materials and Methods

### 2.1. Materials

Vardenafil (VDF) was provided by G.N.P. Co. (6th of October City, Giza, Egypt). Cholesterol, hydroxypropyl cellulose (100,000 cps), sodium deoxycholate (SDC), and soybean phosphatidylcholine (SPC) were purchased from Sigma Aldrich (St. Louis, MO, USA). All other chemicals and reagents used were of analytical grade.

### 2.2. Animals

Male albino rats weighing from 180–200 g were maintained in a humidity- and temperature-controlled environment, with free access to laboratory chow and water. The animal study protocol was authorized by Ethical Committee, College of Pharmacy, Prince Sattam Bin Abdulaziz University, Al-Kharj, KSA (approval number: 048/2022).

### 2.3. Preparation of Vardenafil-Loaded Bilosomes 

Thin-film hydration technique was adopted for the preparation of vardenafil (VDF)-loaded bilosomes, composing of soybean phosphatidylcholine (SPC), cholesterol (CHOL), and sodium deoxycholate (SDC) [23]. In brief, definite amounts of SPC, CHOL, and VDF were thoroughly dissolved in a mixture of chloroform–methanol (1:1, *v*/*v*). A rotary evaporator was used to vaporize the organic solvent at 60 °C under reduced pressure to generate a thin lipid layer. An amount of 25 mL of distilled water containing a definite amount of sodium deoxycholate (SDC) was then added to hydrate the formed lipid film. The formed vesicles were then sonicated for 3 cycles of 5 min with a 5-min interval using a UltraSonicator (Hielscher, Teltow, Germany) to attain the required vesicle size of VDF-loaded bilosomes. The bilosomal dispersions were kept in a refrigerator at 4 °C until further experiments.

### 2.4. Experimental Design 

A 3-factor, 3-level Box–Behnken design (3^3^ BBD) with three central points was employed for the preparation and optimization of VDF-loaded bilosomes, and to scrutinize the impact of various formulation variables on bilosomal characteristics. Table 1 summarizes the list of independent formulation variables. The independent variables studied were: (X_1_) SPC molar concentration; (X_2_) CHOL molar concentration; and (X_3_) amount of SDC. All these formulation variables were studied at three levels, described as low (−1), medium (0), and high (+1). A total of 15 formulae were prepared (Table 2). The impact of such formulation variables on two product responses, particle size (Y_1_) and percentage entrapment efficiency (Y_2_), was explored. The significance of each factor was analyzed by ANOVA using Design-Expert^®^ software version 12 (Stat-Ease, Inc., Minneapolis, MN, USA).

### 2.5. Characterization of VDF-Loaded Bilosomes

#### 2.5.1. Transmission Electron Microscope (TEM)

A Transmission Electron Microscope ((Joel JEM 1230, Tokyo, Japan) was adopted to visualize the surface morphology of VDF-loaded bilosomes. In brief, one drop of properly diluted VDF-loaded bilosomal dispersion was adsorbed onto a carbon-coated copper grid, stained with 1% phosphotungstic acid and air dried before being examined by TEM at an accelerating voltage of 100 kV.

#### 2.5.2. Determination of Particle Size

The mean particle size, polydispersity index, and zeta potential of the prepared VDF-loaded bilosomes were obtained using a laser scattering particle size analyzer (Malvern Instrument, Malvern, UK). An amount of 100μL of bilosomal dispersion was subjected to suitable dilution with distilled water and the measurements were taken in triplicate at 25 °C.

#### 2.5.3. Entrapment Efficiency (EE %) 

The entrapment efficiency (EE%) of VDF within bilosomes was determined indirectly by measuring the free non-encapsulated VDF in the bilosomal dispersion. Briefly, 1 mL of VDF-loaded bilosomes was centrifuged at 15,000 rpm at 4 °C for 1 h. The concentration of free VDF in the separated supernatant was estimated spectrophotometrically at λ_max_ 270 nm using UV–Visible spectrophotometer (Shimadzu, model UV-1601 PC, Tokyo, Japan). The following formula was used to calculate the EE%:

(1) $$EE\;\left(\%\right)=\frac{Total\;initial\;amount\;of\;VDF\;-\;amount\;of\;free\;VDF}{Total\;initial\;amount\;of\;VDF}\;\times \;100$$

#### 2.5.4. Differential Scanning Calorimetry (DSC)

Thermal properties of VDF, SPC, CHOL, SDC, and optimized VDF-loaded bilosomal formulation were scanned utilizing a differential scanning calorimeter (Shimadzu DSC-50, Tokyo, Japan). Briefly, 3 mg of each sample were enclosed in an aluminum pan and heated to temperatures ranging from 10 to 300 °C at a continuous heating rate of 10 °C/min under a nitrogen atmosphere [24].

### 2.6. In Vitro Drug Release Study

In vitro release of VDF from VDF-loaded bilosomal dispersion was estimated using bag diffusion method [7]. Briefly, a dialysis tubing cellulose membrane (MW cut-off 12,000–14,000 Da), immersed overnight in release medium, containing adequate volume of VDF-loaded bilosomal dispersion (equivalent to 2 mg VDF) was suspended in 250 mL of release medium. The release medium consisted of phosphate-buffered saline (PBS; pH 6.8) containing 0.5% sodium lauryl sulfate, stirred at 100 rpm for 24 h and maintained at 37 ± 1 °C. At scheduled time points, 2 mL samples were collected from the release medium and replenished with 2 mL of fresh release media. The concentration of VDF in each sample was quantified spectrophotometrically at λ_max_ 270 nm.

### 2.7. Stability Studies for Vardenafil-Loaded Bilosomes

Stability testing of the optimized VDF-loaded bilosomal formulation was conducted by storing bilosomal dispersion at 4 ± 1 °C in glass vials for 3 months. The change in entrapment efficiency and/or particle size before and after storage time were used as markers of physical stability of bilosomal formulation [25].

### 2.8. Preparation of Vardenafil-Loaded Bilosomal Sponge

The optimized formula of VDF-loaded bilosomes were incorporated into a cellulose-based matrix consisting of hydroxypropyl cellulose/carboxymethyl cellulose aqueous blend, in a 50:50 ratio, by mechanical stirring for 4 h until obtaining a homogenous mixture. The resulting mixture was then put into a 1 cm diameter mold and lyophilized for 24 h. 

### 2.9. Characterization of Vardenafil-Loaded Bilosomal Sponge

#### 2.9.1. pH

VDF-loaded bilosomal sponge (1 cm diameter) was allowed to swell for 2 h at room temperature with 3 mL of simulated saliva fluid (SSF; pH 6.8). Then, the pH of VDF-loaded bilosomal sponge was assessed by a pH-meter (Adwa, Hungary). Each sample was tested in triplicate.

#### 2.9.2. Drug Content Determination

Accurately weighed amount of bilosomal sponge (equivalent to 2 mg VDF) was dissolved in 10 mL PBS and drug content was analyzed at λ_max_ 270 nm. The percentage of drug content was estimated using the following equation:

(2) $$Drug\;content\;\left(\%\right)=\frac{Actual\;amount\;of\;drug\;in\;formulation}{Theoretical\;amount\;of\;drug\;in\;formulation\;}\;\times \;100\;\;\;\;\;$$

#### 2.9.3. Ex Vivo Mucoadhesion Time

Mucoadhesiveness evaluation was conducted by evaluating the mucoadhesive time of the prepared VDF-loaded bilosomal sponge to sheep buccal mucosa. Briefly, VDF-loaded bilosomal sponge (1 cm diameter) was moistened with 100 μL SSF (pH 6.8) and attached to sheep buccal mucosa fixed onto a glass side of a container holding 100 mL SSF (pH 6.8), stirred at a rate of 50 rpm [7]. The complete detachment of the formulation from the sheep buccal mucosa was taken as an endpoint for the estimation of mucoadhesive time [26].

### 2.10. Ex Vivo Permeation Study

Ex vivo permeation of VDF from VDF-loaded bilosomal sponge was examined using sheep buccal mucosa. In brief, sheep buccal mucosa was attached to one end of an open-ended glass tub and a specified weight of VDF-loaded bilosomal sponge (corresponding to 2 mg VDF) was placed. The tube was then fixed to the shaft of a dissolution apparatus I. The dissolution apparatus was supplemented with 250 mL of PBS (pH 6.8) containing 0.5% sodium lauryl sulfate as a permeation medium and was stirred at 100 rpm at 37 °C for 24 h. At scheduled time points, aliquot samples (2 mL) were collected from the permeation medium and replenished with 2 mL of fresh media. The concentration of VDF in each sample was quantified spectrophotometrically at λ_max_ 270 nm. A similar experiment was carried out using plain VDF gel containing 2 mg of drug. Permeation parameters such as the flux at 24 h (J_max_) and the enhancement ratio (ER) were computed using the following equations:

(3) $$Jmax=\frac{amount\;of\;permeated\;drug}{Time}$$

(4) $$ER=\frac{J_{max}\;of\;formulation}{J_{max}\;of\;plain\;gel}$$

### 2.11. In Vivo Studies

#### 2.11.1. Pharmacokinetic Study

Albino rats were divided into two groups (n = 6); the control group was treated orally with plain vardenafil (20 mg/kg), while the other group was treated with VDF-loaded bilosomal buccal formulation (20 mg VDF/kg). At definite time points post-treatment, 200 μL blood samples were withdrawn from the rats in heparinized tubes and centrifuged at 4000 rpm for 15 min. The drug concentration in the separated plasma samples was determined by HPLC analysis. In brief, an HPLC (Shimadzu, Tokyo, Japan) equipped with C18 (150 mm × 4.6 mm, 5 μm) column was used. The mobile phase consisted of 0.1 orthophosphoric acid and methanol acetonitrile and water (40:60% *v*/*v*). The flow rate was 1.0 mL/min. The detection was carried out at 230 nm, with an injection volume of 20 µL. PKSolver 2.0 software was used to determine VDF pharmacokinetic parameters, such as maximum plasma concentration (C_max_), half-life (t_1/2_), area under the plasma concentration-time curve (AUC_0-t_), and median residence time (MRT).

#### 2.11.2. Measurement of cGMP Level in Serum

The rats were randomly grouped into three groups (n = 6). A negative control group received phosphate buffer saline via gastric gavage. The second group was treated with an oral VDF solution (20 mg/Kg), while the third group was treated with VDF-loaded bilosomal buccal formulation (20 mg VDF/kg). At 2, 4, and 6 h post-treatment, 200 μL blood samples were collected from each rat. To obtain serum, blood samples were kept aside at 25 °C for 30 min, and centrifuged for 15 min at 5000 rpm. The serum cGMP levels were quantified using a cGMP Direct Immunoassay Kit (Abcam, MA, USA) in accordance with manufacturer instructions.

### 2.12. Statistical Analysis

All values represent mean ± standard deviation. An unpaired Student’s *t*-test and one-way ANOVA (GraphPad Software, San Diego, CA, USA) were employed for statistical significance analysis. A *p* < 0.05 indicates the presence of a significant difference.

## 3. Results

### 3.1. Experimental Design and Optimization

Box-Behnken design (BBD) is a commonly used response surface methodology adopted to develop higher order response surfaces using fewer required runs than a normal factorial design. In the current study, a three-level, three factor BBD was employed for the fabrication and optimization of vardenafil-loaded bilosomes. The variables listed and their levels were designated based on exploratory experiments to find out the probable independent factors (Table 1). A total of 15 formulations, with three central points (Table 2), were obtained by altering three formulation parameters; soybean phosphatidylcholine (SPC) molar concentration (X_1_), Cholesterol (CHOL) molar concentration (X_2_), and sodium deoxycholate (SDC) amount (X_3_). The effect of these formulation variables was examined on two formulation characteristics: namely, particle size (Y_1_) and percentage entrapment efficiency (%EE, Y_2_). 

### 3.2. Impact of Formulation Variables on Product Characteristics of VDF-Loaded Bilosomes

#### 3.2.1. Effect of Formulation Variables on Particle Size

**Figure 1:**
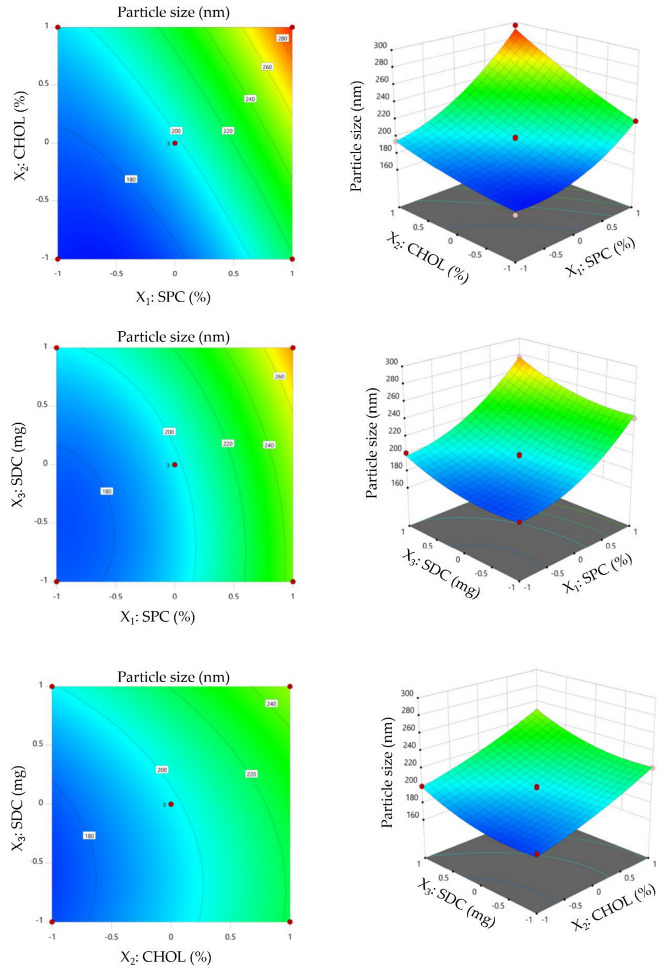
Contour and 3D surface plots showing the influence of independent variables (SPC, CHOL, and SDC) on bilosomal size.

Particle size of nanovesicles is a key determinant for drug absorption. Generally, decreasing particle size is crucial for promoting drug penetration through mucosal membranes. The mean particle size of the prepared VDF-loaded bilosomes fluctuated from 165.8 ± 9.3 to 296.1 ± 10.8 nm (Table 2). The significance and impact of the tested independent variables (SPC molar concentration, CHOL molar concentration, and SDC amount) on particle size of VDF-loaded bilosomes were tested by ANOVA analysis (Table S1) and depicted graphically by contour plots and 3D response surface graphs (Figure 1). As depicted in Figure 1, SPC molar concentration, CHOL molar concentration, and SDC amount exerted a significant positive effect on the particle size of VDF-loaded bilosomes. At fixed cholesterol concentrations, increasing the lipid amount from 1 to 3% resulted in a considerable rise in the size of VDF-loaded bilosomes. The mean particle size of F2 at 3% lipid concentration (241.3 ± 9.8 nm) was significantly larger than that of F12 (178.4 ± 7.9 nm) or F11 (199.5 ± 8.4 nm), prepared at 1 and 2% lipid concentration, respectively. Similar findings were stated by Ahmed et al., who revealed that increasing the concentration of lipids from 0.02 to 0.06 M triggered a remarkable increase in the vesicle size of lornoxicam-loaded bilosomes [27].

Similarly, the inclusion of cholesterol within VDF-loaded bilosomes caused a significant rise in vesicles size. At constant SPC concentration, bilosomal formulation (F13) prepared with 30% cholesterol showed significantly larger particle size than that lacking cholesterol (F7). The particle sizes of F7 and F13 were 199.6 ± 10.2 nm and 247.6 ± 7.1 nm, respectively. These findings might be ascribed to the fact that increasing cholesterol concentration will trigger more cholesterol molecules to be dispersed into the phospholipid bilayer, resulting in a remarkable increase in the bilosomal particle size.

Notably, increasing the SDC amount was found to trigger a significant increase in the vesicle size of VDF-loaded bilosomes. Vesicle size of bilosomes fabricated with 30 mg SDC was significantly higher than that prepared with 10 mg SDC (247.6 ± 7.1 nm (F13) vs. 221.1 ± 11.2 nm (F1)). Bile salts are negatively charged. Consequently, increasing the concentration of SDC would result in an increased repulsion between the vesicles bilayers, resulting in a rise in particle size [28]. Furthermore, the bulkiness effect of SDC imparted by its steroid-like nature could trigger the rise in PS [29].

A BBD-derived mathematical equation validated the positive impact of SPC molar concentration (X_1_), CHOL molar concentration (X_2_), and SDC amount (X_3_) on the detected response (Y_1_; particle size). Where, the positive coefficient refers to a synergistic action of independent variables on tested dependent response:

Y_1_ = 197.37 + 36.49 X_1_ + 24.62 X_2_ + 13.19 X_3_ + 12.2 X_1_X_2_ + 2.97 X_1_X_3_ + 1.35 X_2_X_3_ + 16.82 X_1_^2^ + 4.20 X_2_^2^ + 9.77 X_3_^2^

#### 3.2.2. Impact of Formulation Variables on Entrapment Efficiency

**Figure 2:**
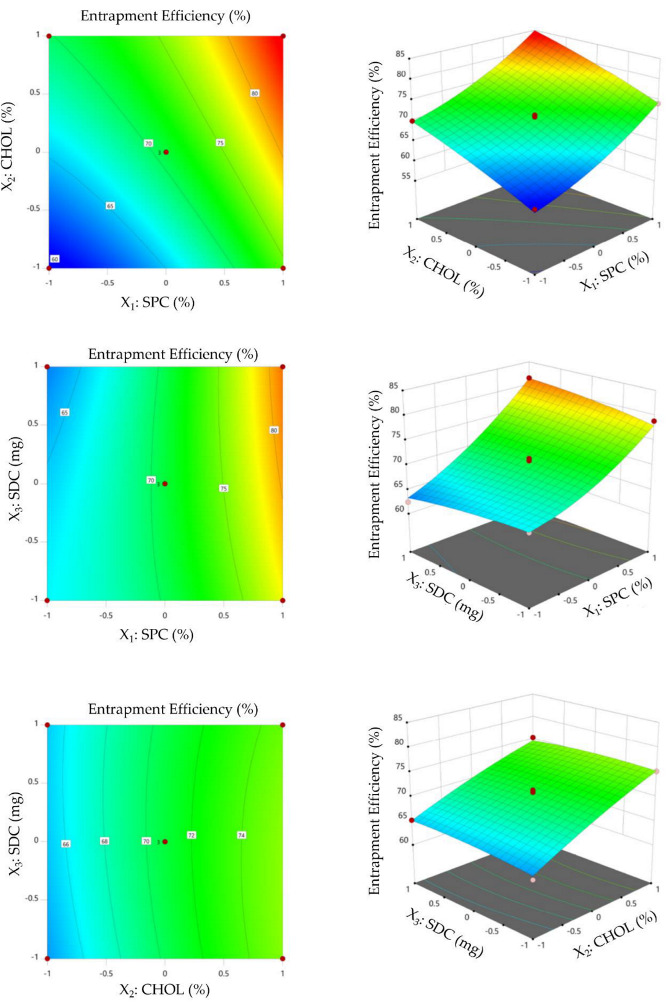
Contour and 3D surface plots showing the influence of formulation variables (SPC, CHOL, and SDC) on entrapment efficiency (%).

The capacity of bilosomes to encapsulate large amounts of drug is a remarkable feature that leads to its potential implementation as an oral delivery device. The entrapment efficiency (EE %) ranged from 60.31 ± 4.1% to 84.14 ± 7.5%. The impact of various formulation variables on entrapment efficiency (EE%) of VDF-loaded bilosomes is depicted graphically (Figure 2). It was evident that raising the concentration of SPC significantly increased the entrapment efficiency percentage. The EE% of bilosomes prepared at a 1% molar concentration of SPC (F4) showed a significantly lower entrapment efficiency % compared to those prepared with a 3% SPC (F9). The EE% of F4 was 69.98 ± 4.7%, while that of F9 was 84.14 ± 7.5%. The remarkable rise in entrapment efficiency with increasing SPC concentration might be ascribed to the mutual increase in particle size with increasing SPC. In the same context, it was evident that incorporating cholesterol into bilosomal membrane significantly enhanced entrapment efficiency. Bilosomal formulation prepared with 30% molar concentration of cholesterol (F1) showed a remarkable increase in EE% (75.25 ± 6.9%), compared to that prepared without cholesterol (F10; EE% was 63.20 ± 4.2%). It has been demonstrated that cholesterol increases the hydrophobicity and rigidity of bilosomal bilayer membranes. As a consequence, it increases bilosome stability and prevents entrapped drug leakage [30,31]. Similar findings were stated by Elkomy et al. [32], who revealed that the entrapment of the alkaloid nutraceutical berberine into bilosomes was significantly enhanced by increasing the concentration of both SPC and CHOL. Notably, ANOVA analysis (Table S1) revealed that changing the amount of SDC exerted an insignificant effect on the entrapment efficiency of VDF within bilosomal formulations. Interestingly, our findings were validated by the fitted mathematical polynomial equation derived from the BBD, which demonstrated the synergistic influence of SPC concentration (X_1_) and CHOL concentration (X_2_) on entrapment efficiency (Y_2_).

Y_2_ = 70.85 + 7.49 X_1_ + 5.23 X_2_ + 0.13 X_3_ + 0.07 X_1_X_2_ + 1.56 X_1_X_3_ − 0.46 X_2_X_3_ + 1.90 X_1_^2^ − 0.59 X_2_^2^ − 0.49 X_3_^2^

#### 3.2.3. Selection of Optimized VDF-Loaded Bilosomal Formulation 

A mathematical optimization technique was adopted to identify the optimized formula that met the criteria of having maximum EE while keeping the vesicle size within the nano-range. The composition of the optimized VDF bilosomal formulation derived from numerical optimization was SPC 2.98%, CHOL 29.4%, and SDC 17.25 mg, with desirability approaching 1. To validate the optimization process, the proposed optimized formula was prepared and assessed for particle size (nm) and entrapment efficiency (%) for the purpose of comparison with the predicted responses. The measured particle size and entrapment efficiency (%) were 282.6 ± 9.5 nm, and 82.95 ± 3.5%, respectively, which were comparable to the predicted values (284.7 nm and 84.19%) for the optimized formula. These findings ensured the validity of the optimization approach for the formulation of VDF-loaded bilosomes based on 3^3^ BBD.

### 3.3. Characterization of Optimized VDF-Loaded Bilosomes

#### 3.3.1. Particle Size, Zeta Potential, and Poly Dispersity Index

**Figure 3:**
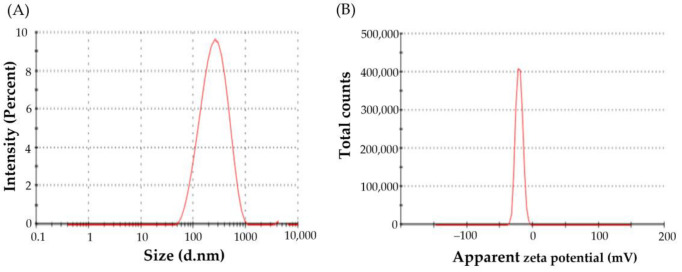
(**A**) DLS size distribution plot; (**B**) zeta potential.

Vesicle size is a key determinant of the rate and extent of drug release, and the in vivo fate of nanocarriers as well. The average vesicle size of the optimized VDF-loaded bilosomes was 282.6 ± 9.5 nm, as evaluated by DLS (Figure 3A). The polydispersity index (PDI) is another crucial parameter that depicts the homogeneity of colloidal systems. Generally, colloidal systems with PDI values less than 0.3 are considered homogenous [33]. In the current study, the PDI of optimized VDF-loaded bilosomes was 0.269, indicating homogenous size distribution.

Another crucial parameter that dictates the colloidal stability of nanoparticles is the zeta potential. Generally, colloidal systems with high zeta potential values ranging from −20 to +20 mV are considered stable, due to the generated electrostatic repulsion between the adjacent nanoparticles. Herein, the zeta potential of the prepared optimized VDF-loaded bilosomes was −20.4 ± 1.2 mV (Figure 3B), suggesting good stability of the formulated nanovesicles.

#### 3.3.2. Transmission Electron Microscopy

**Figure 4:**
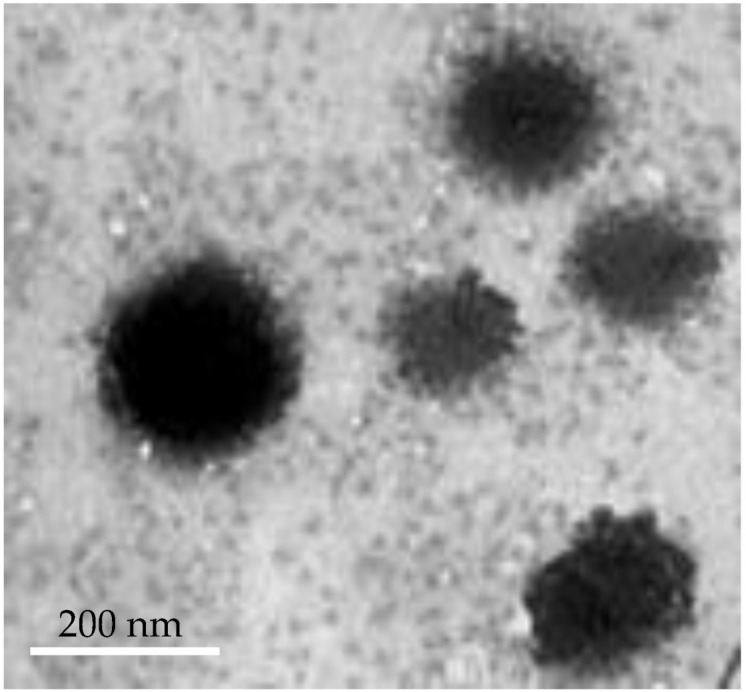
Transmission electron imaging of optimized VDF-loaded bilosomal formulation.

The transmission electron microscopy (TEM) method is an effective tool for obtaining comprehensive information concerning the structural properties of nanoscale vesicles. TEM imaging of optimized VDF-loaded bilosomes depicts that the optimized formula was well-identified spherical vesicles with a relatively smooth surface (Figure 4).

#### 3.3.3. Differential Scanning Calorimetry (DSC)

**Figure 5:**
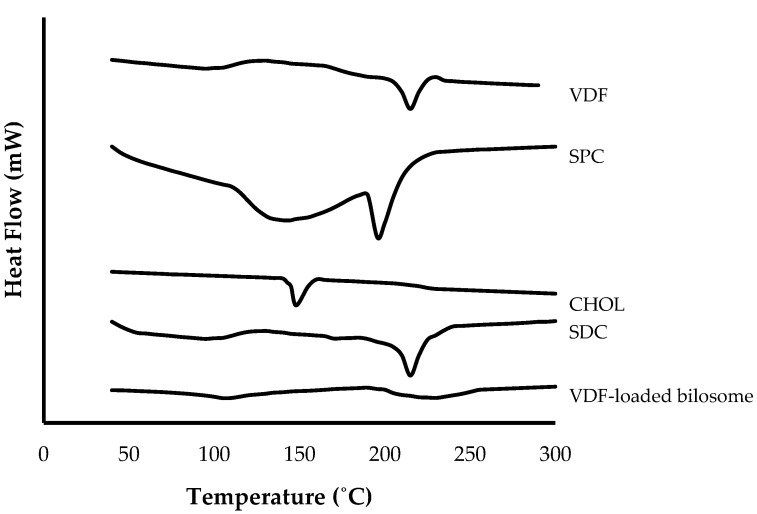
DSC thermograms of pure VDF, soybean phosphatidylcholine (SPC), cholesterol (CHOL), sodium deoxycholate (SDC), and optimized bilosomal formulation.

Differential scanning calorimetry (DSC) is a thermal analysis technique that was adopted to assess the physical characteristics and purity of VDF sample along with its interaction with excipients. Thermal properties of VDF, SPC, CHOL, SDC, and optimized VDF-loaded bilosomal formulation were recorded utilizing differential scanning calorimeter (Figure 5). The pure vardenafil (VDF) thermogram exhibited a broad endothermic peak from 50–100 °C, corresponding to water loss, and another characteristic endothermic peak at 118 °C, corresponding to VDF melting [34]. The SPC thermogram showed an endothermic peak at 167 °C. The thermograms of CHOL and SDC exhibited endothermic peaks at 146 °C and 215 °C, respectively. Intriguingly, no characteristic endothermic peaks were identified for optimized VDF-loaded bilosomal formulation, indicating that VDF was entirely encapsulated within bilosomes.

### 3.4. In Vitro Release Study

**Figure 6:**
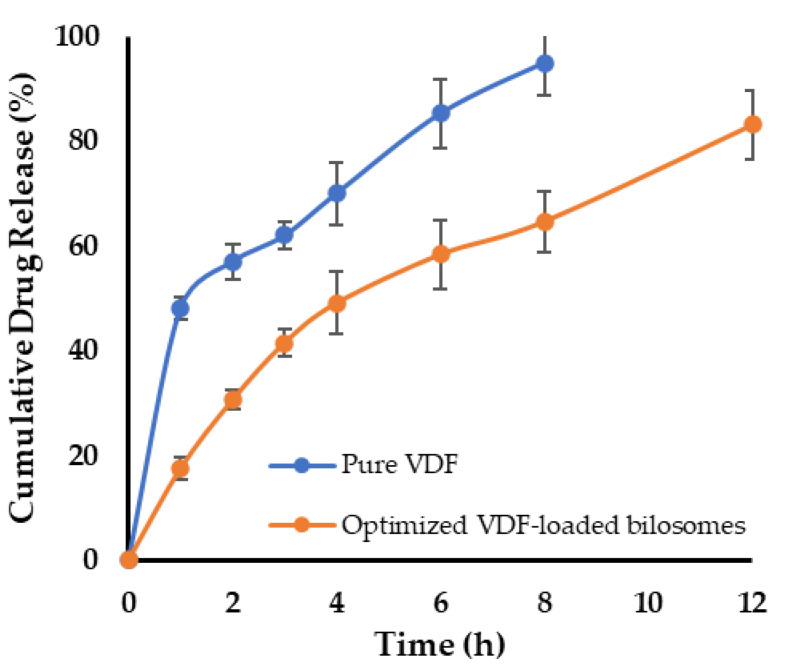
In vitro release profile of VDF-loaded bilosomes. Data represent mean ± SD of three independent experiments.

Figure 6 depicts the in vitro release/dissolution pattern of free VDF and optimized VDF-loaded bilosome. Drug release rate from VDF suspension was substantially greater than that from the optimized bilosomal formulation (*p* 0.05), with about 60% of the drug released within 2 h. In contrast, drug release from optimized VDF-loaded bilosomes was found to be much slower than free drug; with a maximum of 30% at 2 h and less than 60% at 6 h (Figure 6). The relatively slower drug release of VDF from VDF-loaded bilosomes, compared to free drug, might be ascribed to the higher affinity of poorly water-soluble VDF to the hydrophobic components of bilosomes. Nevertheless, the cumulative percentage release of VDF from bilosomes reached more than 80% at the end of 12 h, suggesting that entrapment of VDF within bilosomal formulation could sustain drug release for a prolonged time, compared to free drug.

### 3.5. Stability Studies

Understanding and controlling the stability of colloidal dispersions is crucial for its satisfactory use. Herein, following 90 days of storage at 4 ± 0.5 °C, no signs of aggregation or change in appearance of optimized VDF-loaded bilosomal formulation were observed. Additionally, there were no significant changes in the particle size, zeta potential, or entrapment efficiency of the stored formulation, compared to freshly prepared formula (Table S2). Such storage stability of VDF-loaded bilosomes might be ascribed to the negatively charged nature of bilosomes imparted by SDC, which hinders the fusion or aggregation of the stored bilosomal vesicles [29].

### 3.6. Preparation of VDF-Loaded Bilosomal Mucoadhesive Sponge

Solvent casting method was adopted for the preparation of bilosomal VDF-loaded sponge using an aqueous blend of hydroxypropyl cellulose/carboxymethyl cellulose (HPMC/CMC) as a polymer-forming matrix. 

### 3.7. Evaluation of the Developed VDF-Loaded Bilosomal Sponge

VDF-loaded bilosomal sponge was examined for pH, drug content, and mucoadhesive time. The pH of the formulated bilosomal sponge was 6.42 ± 0.19, which is thought to be suitable with buccal mucosa [35], eliminating the likelihood of buccal mucosal discomfort during application. Drug content of the formulated VDF-loaded bilosomal sponge was 98.15 ± 1.1%, revealing the efficient drug incorporation with the cellulose-based matrix.

Mucoadhesion time is regarded as an important predictor of the effectiveness of medications intended for buccal delivery [7]. Extending the mucoadhesive time in the buccal cavity permits intimate contact with buccal mucosa, triggering better drug absorption. Herein, no detachment of VDF-loaded bilosomal formulation from sheep buccal mucosa was detected for up to 3 h. Such a relatively long mucoadhesion time is thought to be appropriate for contact with buccal mucosa, assisting the wetting of the dosage form, and therefore facilitating effective VDF release from bilosomal formulation.

### 3.8. Ex Vivo Permeation Study through Sheep Buccal Mucosa

**Figure 7:**
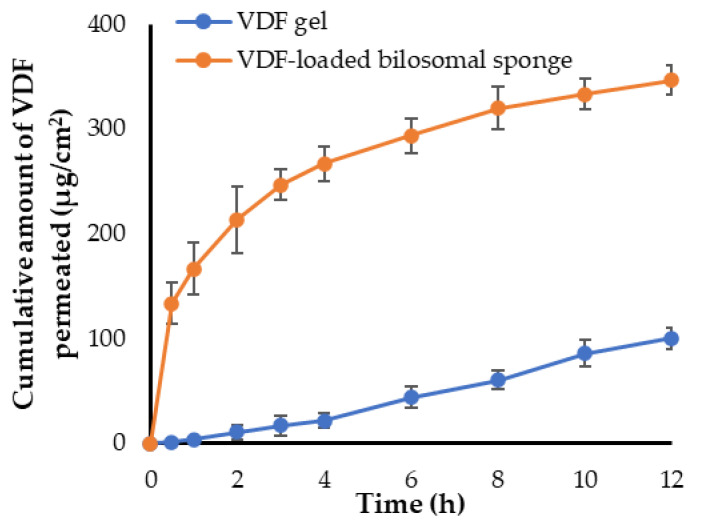
Ex vivo permeation of VDF-loaded bilosomal sponge from sheep buccal mucosa.

The ex vivo permeation profile of VDF from optimized VDF-loaded bilosomal sponge through sheep buccal mucosa is depicted in Figure 7. It was evident that the VDF-loaded bilosomal sponge penetrated more VDF after 12 h, than the VDF gel. The total amount of drug permeated at 12 h was 346.67 ± 14.2 μg/cm^2^ for VDF-loaded bilosomal sponge, compared to 100.13 ± 9.9 μg/cm^2^ for VDF gel. In addition, VDF-loaded bilosomal formulation revealed greater flux (J_max_ of the VDF-loaded bilosomal sponge = 28.88 ± 3.1 μg/cm^2^/h) compared to (J_max_ of VDF gel = 8.34 ± 1.1 μg/cm^2^/h). This remarkable increase in bilosome flux may be ascribed to the high phospholipid content and small vesicular size of VDF-loaded bilosomes, which increases bilosome affinity to biological membranes, and thereby increasing permeation [36]. In addition, bile salts have been reported to negatively affect the integrity of the tight junction of membranes [15]. Consequently, the incorporated SDC within VDF-loaded bilosomes could enhance drug permeation via promoting the opening of the tight junction of the mucosal membrane. Interestingly, the enhancement ratio (ER) of optimized bilosomal formulation was 3.46, suggesting the efficacy of bilosomal formulation to enhance entrapment and permeation of VDF. Table 3 summarizes the results of several permeation parameters calculated through sheep buccal mucosa. 

### 3.9. In Vivo Study

#### 3.9.1. Bioavailability and Pharmacokinetic Study

**Figure 8:**
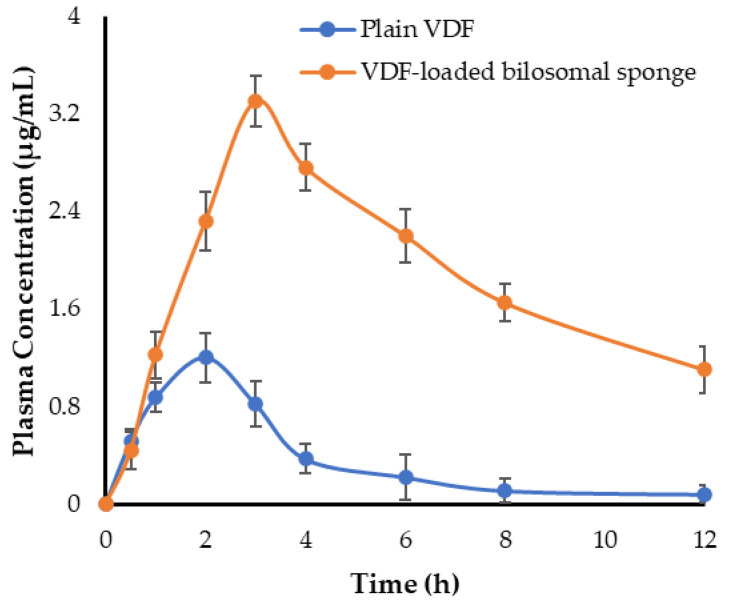
Plasma concentration of VDF following treatment with VDF-loaded bilosomal buccal sponge and VDF oral suspension. Data represented as mean ± SD.

The pharmacokinetic profiles of oral VDF suspension and VDF-loaded buccal bilosomal formulation are represented in Figure 8. As shown in Figure 8, plain VDF plasma levels were very low, owing to its limited water solubility, which may have hampered its effective absorption. In contrast, animals treated with VDF-loaded bilosomal buccal sponge showed much higher plasma concentrations of VDF, suggesting a dramatic increase of drug absorption from bilosomal buccal sponge. The key pharmacokinetic parameters of plain VDF and VDF-loaded bilosomal buccal sponge are summarized in Table 4. For plain VDF, C_max_ and AUC_0-t_ were 1.2 ± 0.18 μg/mL and 4.41 ± 0.65 μg.h/mL, respectively. VDF-loaded bilosomal buccal sponge, on the other hand, delivered a significantly higher VDF plasma level and sustained it for a long time. C_max_ and AUC_0-12_ values were 3.3 ± 0.21 μg/mL and 22.44 ± 1.35 μg.h/mL, respectively. These results revealed that formulating VDF into bilosomal buccal sponge significantly improved VDF oral absorption. Such remarkable increase in oral absorption of VDF from bilosomal formulation, compared to plain drug, might be attributed, at least in part, to the efficient entrapment of the drug into nanoscale vesicles with improved permeability, along with bypassing first-pass metabolism of the drug via its buccal delivery [37]. In addition, compared to plain VDF, VDF-loaded bilosomal buccal formulation significantly (*p* < 0.05) extended VDF residence time in the blood circulation. The MRT of plain VDF and VDF-loaded bilosomal buccal sponge were 4.17 ± 0.51 h and 10.03 ± 0.82 h, respectively. Such an increase in MRT of VDF-loaded bilosomal formulation might be attributed to the gradual and extended release of VDF from bilosomal sponge along with the reduced clearance of VDF from the systemic circulation. Most importantly, the relative bioavailability of VDF-loaded bilosomal buccal formulation was ~5-fold that of the plain VDF. This improved bioavailability might be assigned to enhanced drug absorption from bilosomal vesicles by buccal mucosa while avoiding hepatic first-pass metabolism.

#### 3.9.2. Serum Level of cGMP

**Figure 9:**
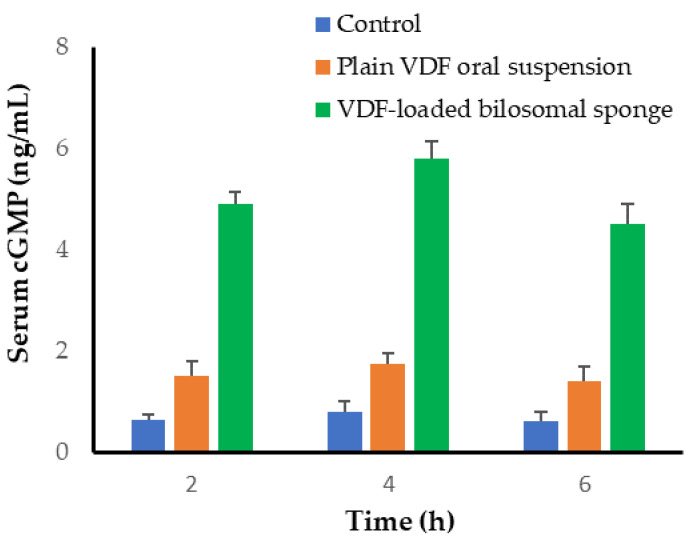
Serum cGMP levels following treatment with VDF-loaded bilosomal buccal sponge and VDF oral suspension. Data represented as mean ± SD.

The cyclic guanosine monophosphate (cGMP) is a cellular regulatory agent that modulates various downstream effects, including neurotransmission, calcium homeostasis, and vasodilation [38]. Elevation of cGMP within smooth muscle leads to relaxation and allows increased blood flow [39]. Vardenafil is a selective inhibitor of cGMP-specific PDE-5 enzyme that competitively inhibits cGMP degradation by PDE-5 enzyme, promoting cGMP accumulation, resulting in vascular smooth muscle relaxation and vasodilation [2]. Consequently, to gain a better understanding of the in vivo fate of VDF following buccal administration of VDF-loaded bilosomal formulation, cGMP serum levels were assessed following the administration of either plain VDF suspension or VDF-loaded bilosomal buccal sponge. As shown in Figure 9, compared to normal rats, serum cGMP levels were considerably higher in all treated animals, independent of drug delivery method. Nevertheless, considerably higher levels of serum cGMP were detected in animals treated with VDF-loaded bilosomal buccal sponge, compared to VDF suspension. This higher blood cGMP levels after buccal delivery of VDF-loaded bilosomal formulation, a 3-fold increase, could be credited to the superior drug absorption from bilosomal vesicles, compared to plain VDF. These findings confirm the superior in vivo potential of bilosomal buccal sponge as a viable vehicle for the delivery of the selective oral PDE-5 inhibitor, VDF, compared to plain VDF.

## 4. Conclusions

In this study, a Box-Behnken design was utilized to fabricate and optimize VDF-loaded bilosomes. The optimized bilosomal formulation exhibited high entrapment efficiency, small particle size, and good physical stability. In addition, the optimized bilosomal formulation was successfully incorporated into a cellulose-based matrix. The formulated VDF-loaded bilosomal sponge demonstrated acceptable properties such as acceptable pH and efficient mucoadhesive characteristics that allowed adequate contact time for VDF penetration through buccal mucosa. Most importantly, in vivo investigations demonstrated that VDF-loaded bilosomal buccal formulation could efficiently enhance the pharmacokinetics of VDF and boost its effect on cGMP serum levels, compared to oral VDF suspension. Collectively, this novel bilosome-based VDF delivery vehicle might provide a viable alternative to conventional orally administered VDF formulations, with superior therapeutic efficacy.

## Figures and Tables

**Table 1:** Box-Behnken design used for optimization of VDF-loaded bilosomal formulations.

Independent Variables	Level		
	−1	0	+1
X_1_: SPC molar concentration	1	2	3
X_2_: CHOL molar concentration	0	10	30
X_3_: SDC amount (mg)	10	20	30
Dependent variables	Desirability Constrains		
Y_1_: Particle size (nm)	In range		
Y_2_: Entrapment efficiency (%)	Maximize		

**Table 2:** Composition of the formulated VDF-loaded bilosomal formulations and the obtained responses.

Factors (Independent Variables)				Responses (Dependent Variables)	
	X_1_: SPC (Molar Concentration)	X_2_: CHOL (Molar Concentration)	X_3_: SDC (mg)	Y_1_: Particle Size (nm)	Y_2_: Entrapment Efficiency (%)
F1	2	30	10	221.1 ± 11.2	75.25 ± 6.9
F2	3	10	10	241.3 ± 9.8	78.93 ± 5.1
F3	2	10	20	198.1 ± 13.1	71.43 ± 5.8
F4	1	30	20	194.6 ± 8.9	69.98 ± 4.7
F5	1	0	20	165.8 ± 9.3	60.31 ± 4.1
F6	3	0	20	218.5 ± 7.6	74.21 ± 6.8
F7	2	0	30	199.6 ± 10.2	65.21 ± 5.9
F8	2	10	20	195.6 ± 11.7	70.13 ± 6.3
F9	3	30	20	296.1 ± 10.8	84.14 ± 7.5
F10	2	0	10	178.5 ± 9.5	63.20 ± 4.2
F11	2	10	20	199.5 ± 8.4	70.98 ± 6.3
F12	1	10	10	178.4 ± 7.9	66.13 ± 4.9
F13	2	30	30	247.6 ± 7.1	75.41 ± 6.4
F14	3	10	30	276.2 + 11.2	81.51 ± 6.8
F15	1	10	30	201.4 ± 8.3	62.45 ± 3.9

Data represent mean ± SD of three independent experiments.

**Table 3:** Ex vivo permeation of VDF gel and VDF-loaded bilosomal formulation from sheep buccal mucosa.

Permeation Parameters	VDF Gel	VDF-Loaded Bilosomal Sponge
Total amount of drug permeated (μg/cm^2^)	346.67 ± 14.2	100.13 ± 9.9
J_max_ (μg/cm^2^/h)	28.88 ± 3.1	8.34 ± 1.1
ER	1	3.46

Data represent mean ± SD of three independent experiments.

**Table 4:** Pharmacokinetic parameters of plain VDF (20 mg/kg, p.o.) and VDF-loaded bilosomal buccal formulation (20 mg VDF/kg).

Parameter	VDF Oral Suspension	VDF-Loaded Bilosomal Buccal Sponge
C_max_ (μg/mL)	1.2 ± 0.18	3.3 ± 0.21
T_max_ (h)	2 ± 0.26	3 ± 0.31
T_1/2_ (h)	2.52 ± 0.37	5.98 ± 0.29
K_e_ (h^−1^)	0.275 ± 0.06	0.116 ± 0.04
AUC_0-t_ (μg.h/mL)	4.41 ± 0.65	22.44 ± 1.35
MRT (h)	4.17 ± 0.51	10.03 ± 0.82
Relative bioavailability (%)	-	508.84

Data represent mean ± SD of three independent experiments.

## Data Availability

All data can be found within this article and its supplementary information.

## Supplementary Materials

The following are available online at https://www.mdpi.com/article/10.3390/polym14194184/s1, Table S1: Results of statistical analysis of all dependent variables Y_1_ and Y_2_, Table S2: Stability study for optimized VDF-loaded bilosomal formulation.
