# Supplementary material for: Vardenafil-Loaded Bilosomal Mucoadhesive Sponge for Buccal Delivery: Optimization, Characterization, and In Vivo Evaluation
Source: Polymers (Basel). 2022 Oct 5;14(19):4184. doi: 10.3390/polym14194184 (PMC9573218; doi:10.3390/polym14194184)
Supplement: Supplementary file 1 [file polymers-14-04184-s001.zip › polymers-1945207-supplementary.pdf]

Table S1. Results of statistical analysis of all dependent variables Y_1 and Y_2.

Source	Y_1		Y_2	
	F-Value	*p*-Value	F-Value	*p*-Value
Model	121.42	< 0.0001	78.96	,0.0001
X_1: SPC	617.04	< 0.0001	458.79	,0.0001
X_2: CHOL	281.05	< 0.0001	223.80	,0.0001
X_3: SDC	80.60	00003	0.1463	0.7178
X_1X_2	34.49	0.0020	0.0173	0.9006
X_1X_3	2.05	0.2115	10.02	0.0250
X_2X_3	0.422	0.5444	0.8747	0.3926
X_1^2	60.52	0.0006	13.63	0.0141
X_2^2	3.77	0.1100	1.30	0.3057
X_3^2	20.42	0.0063	0.9140	0.3830
Lack of Fit	6.70	0.1326	3.07	0.2550
R^2 analysis				
R^2	0.9954		0.9930	
Adjusted R^2	0.9872		0.9804	
Predicted R^2	0.9328		0.9053	
Adequate Precision	36.0309		31.5056	

Table S2. Stability study for optimized VDF-loaded bilosomal formulation

Parameter	Fresh	After 3-month storage at (4 ± 0.5 °C)
Particle size (nm)	282.6 ± 9.5	291.4 ± 13.6
Zeta potential (mV)	-20.4 ± 1.2	-19.7 ± 0.9
Entrapment efficiency (%)	82.95 ± 3.5	80.17 ± 4.2

Data represent mean ± SD of three independent experiments.
